# B7-H1 enhances proliferation ability of gastric cancer stem-like cells as a receptor

**DOI:** 10.3892/ol.2015.2949

**Published:** 2015-02-10

**Authors:** YUPENG YANG, KE WU, ENDE ZHAO, WEI LI, LIANG SHI, GENGCHEN XIE, BIN JIANG, YAXIN WANG, RUIDONG LI, PENG ZHANG, XIAOMING SHUAI, GUOBIN WANG, KAIXIONG TAO

**Affiliations:** 1 Department of General Surgery, Union Hospital, Tongji Medical College, Huazhong University of Science and Technology, Wuhan, Hubei 430022, P.R. China; 2 Department of Clinical Laboratory, Union Hospital, Tongji Medical College, Huazhong University of Science and Technology, Wuhan, Hubei 430022, P.R. China; 3 Anesthesiology and Critical Care, Union Hospital, Tongji Medical College, Huazhong University of Science and Technology, Wuhan, Hubei 430022, P.R. China

**Keywords:** B7-H1, cancer stem-like cell, gastric cancer, proliferation

## Abstract

Cancer stem-like cells (CSCs) are a rare tumorigenic population with the ability to self-renew in numerous cancer types. Their existence is considered a pivotal contributor to tumor recurrence. B7-H1 is a ligand of inhibitory inducible co-stimulator (ICOS) that is broadly expressed on various human cancers. ICOS acts as a ligand of programmed death-1 (PD-1) on T cells, induces the immune escape of cancer cells and also acts as a receptor mediating anti-apoptotic effects on cancer cells. However, the expression and function of B7-H1 on CSCs is not yet clear. In the present study, gastric cancer samples were collected and the B7-H1 expression in gastric cancer CSCs was detected. Ki67, a proliferation marker, was found to be expressed at a higher rate in B7-H1^+^ CSCs compared with the B7-H1^−^ counterparts. SGC-7901 cells, a gastric cancer cell line, were cultured in serum-free medium to form sphere cells that possessed stem cell characteristics and could express B7-H1 with the stimulation of interferon-γ. The proliferative ability of sphere cells was enhanced following B7-H1 activation with recombinant PD-1 *in vivo* and *in vitro*. This effect could be eliminated by neutralizing B7-H1. Overall, B7-H1 can act as a stimulating receptor for CSCs, and induce CSC proliferation. Blocking B7-H1 on CSCs may possess therapeutic potential for treating gastric cancer.

## Introduction

Gastric cancer is the second leading cause of cancer-associated death worldwide (1). Current treatments for gastric tumors, including surgery, chemotherapy and radiotherapy, are hampered chiefly by the existence of anti-therapy tumor cells (2). These cells are considered to be cancer stem-like cells (CSCs), which possess self-renewal abilities and can differentiate into all cell types in gastric cancer (3). CSCs have a higher capacity of tumorigenicity and drug-resistance compared with common tumor cells (4). CSCs express numerous stem cell markers, including cluster of differentiation (CD)44, CD166 or Lgr5, in gastric cancer (5,6). Certain cancer cell lines can form spheres when cultured in serum-free medium (7–9). In this condition, sphere cells reveal a high capacity of tumorigenicity and express high stemness-associated genes (7,8). Gastric CSCs (GCSCs) have already been found in gastric cancer cell lines and primary tumors (9–11).

B7-H1 (PD-L1; CD274) is a novel B7 family member that exhibits important suppressive functions in cell-mediated immune responses (12). B7-H1 was observed to be constitutively or inducibly expressed in the majority of human cancers (13). B7-H1 expression in tumor cells was found to be significantly higher compared with normal gastric epithelial cells (14–16). It is well-known that B7-H1 is a ligand of the programmed death-1 (PD-1) receptor, which delivers inhibitory signals to T cells in order to inhibit immune responses (17). B7-H1^+^ tumor cells exhibit increased resistance to CD8^+^ cytotoxic lymphocyte (CTL)-mediated destruction compared with B7-H1^−^ cells (18). Ablation of B7-H1 and PD-1 interaction by blocking antibodies can restore CTL-mediated tumor lysis *in vitro*, suggesting a novel target of cancer therapy (19). Previous studies focused on PD-1 as a receptor that induced T-cell suppression. However, previous evidence has demonstrated that B7-H1 can act as a receptor to transmit signals from T cells to cancer cells. Therefore, it was demonstrated that B7-H1^+^ cancer cells utilize a receptor on immune cells as a ligand to resist apoptosis induced by Fas ligation or the protein kinase inhibitor Staurosporine (20). Notably, constitutive expression of B7-H1 is rare (13), but it can be induced by various inflammatory mediators, including interferon (IFN)-α, -β and -γ, vascular endothelial growth factor, interleukins-4 and -10, and lipopolysaccharide (21–23). Even treating cancer cell lines with antitumor drugs, such as paclitaxel and etoposide, can induce B7-H1 expression (24). Therefore, it is possible that tumor cells express B7-H1 to protect themselves when they are challenged by survival stress. CSCs hamper cancer treatment and are abnormal when facing survival stress. Thus, it was hypothesized that CSCs can express and receive survival signals from B7-H1.

To test the hypothesis, clinical gastric cancer samples were collected using Lgr5 as a CSC marker, and the difference in proliferation status between B7-H1^+^ and B7-H1^−^ cells was compared in GCSC. To explore potential B7-H1 roles in cultured SGC-7901 sphere cells, B7-H1 expression was induced by IFN-γ and recombinant human PD-1 was added to stimulate B7-H1^+^ CSCs. In the present study, it was revealed that B7-H1 acts as a receptor and transmits signals into tumor cells, thereby enhancing the proliferation of GCSCs.

## Materials and methods

### Gastric cancer tissue

A total of 43 surgical specimens of gastric cancer were collected from patients who underwent gastrectomy at the Huazhong University of Science and Technology Tongji Medical College Affiliated Union Hospital (Wuhan, China) between 2012 and 2013. The Institutional Ethical Review Committee of the hospital approved the present study and all patients provided written informed consent.

### Cell culture

The adherent gastric cancer SGC-7901 cell line (SGC-7901-AC) was provided by Dr Wu and was cultured in RPMI-1640 (HyClone, Logan, UT, USA) medium containing 10% fetal bovine serum (FBS; Gibco Life Technologies, Carlsbad, CA, USA) (25). For the spheres, SGC-7901 cells were cultured as previously described (11). All the cells were cultured at 37°C in a 5% CO_2_ atmosphere.

### Colony formation assay

To study the effect of B7-H1 on the proliferation of SGC-7901 sphere cells (SGC-7901-SCs) and the colony-forming ability of adherent and sphere cells, single cells were plated on six-well plates, with 1,000 cells per well, and cultured for two weeks. Following staining with violet, images of the cells were captured and analyzed to assess their proliferative ability. The experiments were performed in triplicate.

### Reverse transcription-quantitative polymerase chain reaction (RT-qPCR)

mRNA extraction and RT-qPCR were performed as previously described (26). Briefly, 500 ng total mRNA was extracted from cells using TRIzol reagent (Takara Bio, Inc., Otsu, Japan) and reversely transcribed to cDNA using PrimeScript RT Master Mix (Takara Bio, Inc.) according to the manufacturer’s instructions. To measure the mRNA levels of genes in each sample, glyceraldehyde 3-phosphate dehydrogenase was used as an internal control. The SYBR Green Master Mix (Takara Bio, Inc.) was used to perform quantitative PCR in the StepOnePlus™ Real-Time PCR system (Applied Biosystems Life Technologies, Foster City, CA, USA). PCR amplification conditions were as follows: Denaturation at 95°C for 30 sec, followed by 45 cycles of 60°C for 60 sec and 95°C for 5 sec. The mRNA expression level for each gene was calculated using the 2^−ΔΔCt^ method. The CD44 primers were as previously described and the other primers used are listed in Table I (9).

### Chemotherapy sensitivity and resistance assay

The cell counting kit (CCK)-8 assay (Dojindo Molecular Technologies, Inc., Kumamoto, Japan) was used to evaluate the sensitivity of gastric cancer cells to chemotherapy, according to the manufacturer’s instructions. Briefly, 5,000 cells were plated into each well of a 96-well plate with growth medium containing various concentrations of fluorouracil (5-FU; Sigma-Aldrich), then incubated at 37°C in 5% CO_2_ for 36 h. The absorbance of each well was recorded at 450 nm using a plate reader. The survival rate (%) was calculated as: (absorbance of experiment − absorbance of blank well)/(absorbance of control − absorbance of blank well) × 100.

### B7-H1 stimulation and blockade in SGC-7901-SC

SGC-7901-SCs were cultured in six-well plates supplemented with recombinant 5 μg/ml PD-1 immunoglobulin (Ig) fusion protein (R&D Systems, Inc., Minneapolis, MN, USA) (27). The B7-H1 blockade was performed by adding specific blocking polyclonal rabbit anti-human PD-L1 antibody (Ab) (H-130; sc-50298; dilution, 1:20; 10 μg/ml; Santa Cruz Biotechnology, Inc., Dallas, TX, USA). The sphere cells were divided into three groups, the control group (PBS), B7-H1 stimulation group (PD-1 Ig, 5 μg/ml) and B7-H1 blockade group (PD-1 Ig, 5 μg/ml; anti-B7-H1 Ab, 10 μg/ml). All groups were treated with IFN-γ (100 ng/ml; PeproTech, Inc., Rocky Hill, NJ, USA) for 24 h. Following IFN-γ treatment, the cells were harvested and washed twice. The proliferation capacity was analyzed using a colony formation assay, xenograft assay in nude mice and flow cytometry.

### Flow cytometry

The gastric cancer tissues were separated into single cells and stained by phycoerythrin (PE)-labeled monoclonal mouse anti-human Lgr5 (clone, 2A2; dilution, 1:50; OriGene Technologies, Inc., Rockville, MD, USA), Alexa Fluor^®^ 488-labeled monoclonal mouse anti-human CD45 (clone:HI30; dilution, 1:20; BioLegend, Inc., San Diego, CA, USA), Per/Cy5.5-labeled monoclonal mouse anti-human CD326 (clone, 9C4; dilution, 1:20; BioLegend, Inc.), APC-labeled mouse monoclonal anti-human B7-H1 (clone:29E.23A; dilution, 1:20; BioLegend, Inc.), PE/Cy7-labeled monoclonal mouse anti-human Ki67 (clone, Ki67; dilution, 1:20; BioLegend, Inc.). The SGC-7901 sphere cells were stained by APC-labeled monoclonal mouse anti-human B7-H1 (clone, 29E.23A; dilution, 1:20; BioLegend, Inc.), Alexa Fluor^®^ 488-labeled monoclonal mouse anti-human Ki67 (clone, Ki67; BioLegend, Inc.) and PE-labeled monoclonal mouse anti-human Lgr5 (clone, 2A2; dilution, 1:50; OriGene Technologies, Inc.). In the tissues, CD326^+^/Lgr5^+^/CD45^−^ cells were classed as GCSCs. In the spheres, Lgr5^+^ cells were classed as CSCs. The Ki67 rate (ratio) = Lgr5^+^/B7-H1^+^ (or B7-H1^−^)/Ki67^+^ cells/total Lgr5^+^/B7-H1^+^ (or B7-H1^−^) cells × 100. The cells were analyzed using a FACSCanto II flow cytometer (BD Biosciences, San Jose, CA, USA).

### Xenograft assay

To investigate the tumorigenicity variation of SGC-7901-SC following treatment with PD-1 Ig fusion protein, 4×10^5^ cells in 250 μl PBS were subcutaneously transplanted into five- to six-week-old male BALB/c nude mice (Beijing HFK Bioscience Co., Ltd., Beijing, China) that weighed 18–22 g (n=5). The animal protocol was approved by the Ethics Committee of Tongji Medical College, Huazhong University of Science and Technology (Wuhan, China). The tumor volumes were recorded every four days using calipers and were determined by the formula: Length × width^2^ ×0.5. At day 32, all mice were euthanized using an i.p. injection of chloral hydrate (2500 mg/kg) and the xenografts were removed and measured.

### Statistical analysis

Each experiment was repeated at least in triplicate. The data are presented as the mean ± standard deviation. The data were analyzed using Student’s t-test and one-way analysis of variance. All statistical analysis was performed using SPSS version 17.0 software (SPSS, Inc., Chicago, IL, USA). P<0.05 was considered to indicate a statistically significant difference.

## Results

### B7-H1^+^ gastric cancer stem cells exhibit an increased proliferative capacity

**Figure 1:**
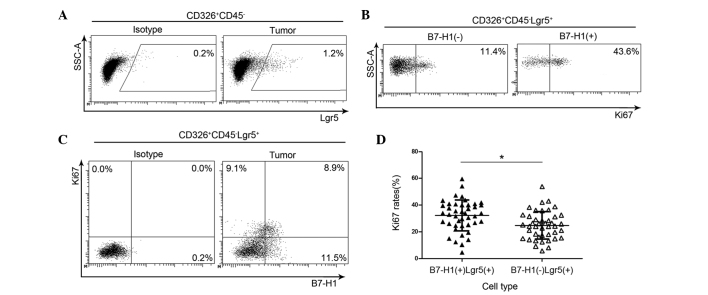
Differences in Ki67 expression between B7-H1^+^ and B7-H1^−^ cells in gastric tissue CSCs. (A) The percentage of Lgr5^+^ gastric cancer stem cells in CD326^+^CD45^−^ gastric cancer tissues. (B and C) Association between B7-H1 and Ki67 in CSCs. The CSCs were divided into B7-H1^+^ and B7-H1^−^ groups. In each group, the Ki67 ratio was revealed. (D) Quantitative analysis of the differences in Ki67 expression rate between B7-H1^+^ and B7-H1^−^ CSCs in gastric cancer tissues. The bars indicated the mean ± standard deviation (n=43). ^*^P<0.05. CSCs, cancer stem cells.

All 43 cases were collected from patients who had not undergone chemotherapy. GCSCs were defined as Lgr5^+^/CD326^+^/CD45^−^ cells and 1.08±0.42% GCSCs were detected in gastric cancer (Fig. 1A). The Ki67 ratio in Lgr5^+^ cells was measured using flow cytometry (Fig. 1B and C). The Ki67 expression rate in Lgr5^+^/B7-H1^+^ cells was 32.22±11.60%, which was significantly higher compared with Lgr5^+^/B7-H1^−^ cells (24.80±10.33%; Fig. 1D), suggesting that B7-H1 may act as a receptor transmitting signals into GCSCs and may also induce GCSC proliferation.

### Generation of sphere cells from gastric cancer cell lines

**Figure 2:**
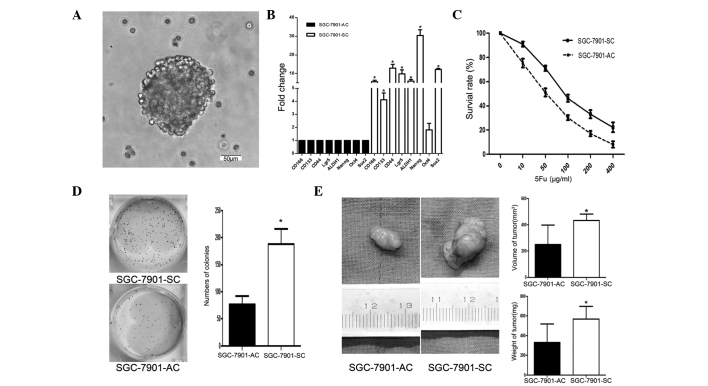
Characteristics of the SGC-7901 sphere cells. (A) Spheres of SGC-7901 cells (bar=50 μm). (B) The expression of the stem core genes CD133, CD166, Lgr5, CD44, ALDH1, Oct4, Nanog and Sox2 in sphere cells were all significantly upregulated in the SGC-7901 sphere cells, with the exception of Oct4. (C) As examined by cell counting kit-8 assay, the survival rates of sphere cells were higher compared with SGC-7901-AC at different 5-Fu concentrations. (D) As evaluated by colony formation assay, the sphere cells demonstrated an improved colony formation ability. (E) As assessed by Xenograft assay in nude mice, the SGC-7901-SCs exhibited improved performance compared with their counterparts (n=5). The bars indicate the mean ± standard deviation. ^*^P<0.05.

**Figure 3:**
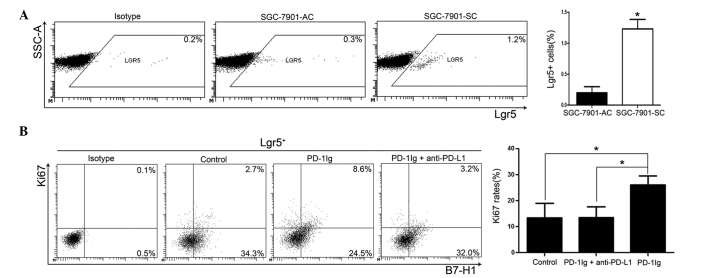
B7-H1 stimulation enhances Ki67 rates in SGC-7901-SC. (A) Cytometric analyses of Lgr5^+^ cells. The percentage of Lgr5^+^ cells was significantly higher in sphere cells. (B) The proportion of Ki67^+^ cells in the stimulation group (26.09±3.50%) was significantly increased compared with the control (13.39±5.61%) and PD-L1 blocking groups (13.56±4.13%). The bars indicate the mean ± standard deviation. ^*^P<0.05.

The ability of the gastric cancer SGC-7901 cell line to form sphere cells in serum-free medium and express B7-H1 under simulation of IFN-γ was explored. SGC-7901 could form spheres (Fig. 2A) and express B7-H1 under the stimulation of IFN-γ (Fig. 3B). Thus, SGC-7901 cells were focused on in the subsequent experiments. For convenience, the SGC-7901 adherent cells and the SGC-7901 sphere cells were designated as SGC-7901-ACs and SGC-7901-SCs, respectively.

### SGC-7901-SCs possess stem cell properties

Initially, the stem core genes were assessed using RT-qPCR and it was found that the expression of Sox2, Nanog, Lgr5, CD166, CD133, ALDH1 and CD44 in SGC-7901-SC was significantly higher compared with SGC-7901-AC, but the expression of Oct4 was not (Fig. 2B), suggesting that SGC-7901-SCs may have a high CSC expression rate.

Subsequently, the chemosensitivity to 5-FU, colony formation and tumor generation were examined in nude mice with SGC-7901-SCs and AC. The present data revealed that the SGC-7901-SCs possessed a higher survival rate compared with SGC-7901-ACs at various concentrations of 5-FU (Fig. 2C).

Using a colony-forming assay and xenograft assay in nude mice, it was found that SGC-7901-SCs retained significantly higher abilities of colony formation (Fig. 2D) and tumor generation (Fig. 2E) compared with SGC-7901-ACs. Finally, the level of Lgr5^+^ cells in SGC-7901-SCs was significantly higher compared with SGC-7901-ACs (Fig. 3A). All these results suggest that SGC-7901-SCs possess stemness properties.

### Stimulation of B7-H1 increases Ki67 levels in B7-H1^+^/Lgr5^+^ CSCs

To investigate the effect of manipulating B7-H1, PD-1 Ig fusion protein was added to activate B7-H1 (27), and anti-B7-H1 Ab was used to block B7-H1 (28). As demonstrated in Fig. 3B, stimulation of B7-H1 significantly increased the level of Ki67 in Lgr5^+^B7-H1^+^ cells in the stimulation group, while anti-B7-H1 treatment significantly decreased the effect. This indicates that B7-H1 acts as a receptor to promote proliferation of CSCs.

### B7-H1 stimulation enhances tumor formation in vitro and in vivo

**Figure 4:**
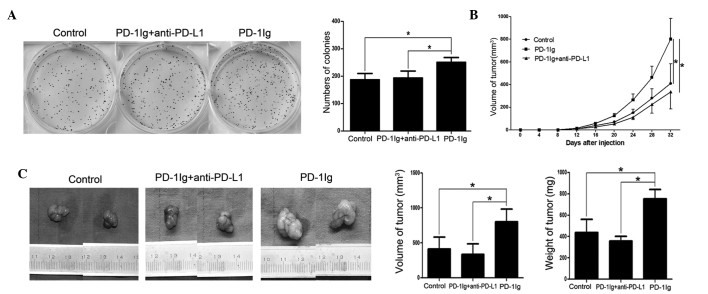
B7-H1 stimulation increases the tumor malignant profile. (A) Groups were divided as same as Fig. 3B, cells in the stimulation group formed significantly more colonies than those in the other groups. The stimulation group demonstrated (B) earlier tumor formation and (C) faster tumor progression compared with the other groups. The bars indicate the mean ± standard deviation. ^*^P<0.05. PD-1, programmed death-1; Ig, immunoglobulin; PD-L1, PD-1 ligand 1.

Colony formation and xenograft assays were performed to investigate the effect of B7-H1 stimulation on GCSCs. The present results revealed that PD-1 Ig treatment significantly increased the colony-forming capacity *in vitro* (Fig. 4A) and mice transferred with PD-1 Ig-treated cells exhibited earlier tumor initiation and faster tumor growth (Fig. 4B and C). Interestingly, tumor initiation and proliferation was significantly inhibited in the mice that received cells treated with B7-H1 blockade (Fig. 4B and C). Overall, the present results indicated that B7-H1 stimulation enhanced the tumor formation of gastric cancer stem cells.

## Discussion

In the present study, CSCs in the SGC-7901 cell line were enriched with serum-free medium. The SGC-7901 cells could form sphere cells in serum-free medium and exhibit characteristics of GCSCs, such as high expression of stem cell core genes (Fig. 2B), exhibiting additional malignant characteristics compared with parental SGC-7901-ACs (Fig. 2C and D) and displaying improved tumor-generating ability when they were subcutaneously transplanted to nude mice (Fig. 2E). Certain gastric cell lines formed sphere cells to enrich CSCs in serum-free medium, but not all cell lines were able to form spheres (9,13).

The present study provided evidence that B7-H1 can act as a receptor to transmit proliferation signals to CSCs. First, by examining the clinical gastric cancer tissue sample, it was found that the Ki67 ratio in B7-H1^+^ CSCs was significantly higher compared with B7-H1^−^ CSCs, which indicated that B7-H1 activation could enhance CSC proliferation (Fig. 1A and B). Second, B7-H1^+^ CSCs in SGC-7901-SCs exhibited elevated Ki67 expression following B7-H1 stimulation. However, the blockade of B7-H1 prevented the Ki67 elevation induced by PD-1 Ig (Fig. 3B) (28). Finally, it was confirmed that the tumor-promoting capability was enhanced by B7-H1 stimulation with *in vitro* colony-forming assay and *in vivo* xenograft assay (Fig. 4A–C). Taken together, the results indicated that B7-H1 acts as a receptor to enhance CSC proliferation.

The majority of previous studies have focused on B7-H1 as a ligand that activates the PD-1 signal pathway and induces T-cell apoptosis, suppression and anergy (13,29,30). However, it was reported that B7-H1 could act as a receptor transmitting signals to cancer cells against therapy (20). Although it appeared that the major mechanism is PD-1 pathway that induces T-cell dysfunction, B7-H1-mediated tumor resistance and PD-1-mediated T-cell dysfunction could work on tumor cell survivals. The existence of CSCs is thought to be an important factor leading to cancer relapse (2). The current study found that CSCs could express B7-H1 and explored a novel perspective of B7-H1 as a receptor to transmit proliferation signals to CSCs. It was reported that PD-1 was widely expressed on T cells, B cells and other immune cells, all of which were found in the tumor microenvironment (20). Therefore, the present findings suggest that CSCs utilize B7-H1 as a receptor to react with PD-1 on immune cells, enhancing their proliferation and self-renewal, which promotes tumor survival.

In previous studies, the application of anti-B7-H1 and anti-PD-1 Ab *in vitro* significantly promoted T-cell activation, proliferation and enhanced cytolytic activity (13,31,32). Therefore, using anti-PD-1 and anti-B7-H1 Abs to treat cancer is promising. The present study used anti-B7-H1 Ab to block B7-H1 on CSCs, and the proliferation effect was fully eliminated. A recent study demonstrated that B7-H1 could bind with CD80 besides PD-1 (33). Since B7-H1 may have more than one receptor and it can enhance proliferation of CSCs, it may not be sufficient to use anti-PD-1 monoclonal Ab alone to treat cancer. The combination of PD-1 and B7-H1 blockade may be promising for tumor therapy. The former restores the cytotoxic ability of T cells against cancer cells, the latter prevents tumor recurrence by inhibiting the proliferation of CSCs.

In conclusion, GCSCs were successfully enriched in the SGC-7901 cell line and it was demonstrated that B7-H1, an inhibitory costimulatory ligand for T cells, could act as a receptor for CSCs to transmit proliferation signals. Therefore, B7-H1 may be a potential target in the treatment of gastric cancer.

## Figures and Tables

**Table I:** Primer names and sequences.

Primer name	5′ to 3′ primer sequence
CD133-F	CTCCCTGTTGGTGATTTGTAT
CD133-R	CCTTGTCCTTGGTAGTGTTGT
CD166-F	TAAACCCAAGTGGAGAAGTGA
CD166-R	CCAGCATCCTGATAATGAAGA
Lgr5-F	AACCTCCTGTCGTCTTTTC
Lgr5-R	GTTGTCACCTTTATTCCAT
Sox2-F	AATGCCTTCATGGTGTGG
Sox2-R	GAGCGTCTTGGTTTTCCG
Oct4	ATGCACAACGAGAGGATTT
Oct4	CAGAGTGGTGACGGAGACA
Nanog-F	GTCTCTCCTCTTCCTTCCT
Nanog-R	TTTTTGCGACACTCTTCTC
ALDH1-F	CGCCAGACTTACCTGTCCTAC
ALDH1-R	TGCCTTGTCAACATCCTCCTT
CD44-F	GCCTTGGCTTTGATTCTTGC
CD44-R	TCCACTTGGCTTTCTGTCCTC
GAPDH-F	CGCTGAGTACGTCGTGGAGTC
GAPDH-R	GCTGATGATCTTGAGGCTGTTGTC
